# Pulmonary Embolism Management Audit and Machine Learning Analysis of Delayed Anticoagulation in a Swiss Teaching Hospital

**DOI:** 10.3390/jcm13206103

**Published:** 2024-10-13

**Authors:** Cedrine Kueng, Maria Boesing, Stéphanie Giezendanner, Jörg Daniel Leuppi, Giorgia Lüthi-Corridori

**Affiliations:** 1 University Institute of Internal Medicine, Cantonal Hospital Baselland, CH-4410 Liestal, Switzerland; cedrine.kueng@ksbl.ch (C.K.); stephanie.giezendanner@ksbl.ch (S.G.); joerg.leuppi@ksbl.ch (J.D.L.); 2 Faculty of Medicine, University of Basel, CH-4056 Basel, Switzerland; 3 Centre for Primary Health Care, University of Basel, CH-4056 Basel, Switzerland

**Keywords:** pulmonary embolism, PE, anticoagulation delay, audit, machine learning, deep vein thrombosis

## Abstract

**Background/Objectives:** Diagnosing acute pulmonary embolism (PE) is challenging due to its wide range of symptoms and numerous differential diagnoses. Medical professionals must balance performing all essential examinations and avoiding unnecessary testing. This study aimed to retrospectively audit the diagnosis and treatment of acute PE at a Swiss public teaching hospital to determine the adherence to current guidelines and to identify the factors associated with the delayed initiation of anticoagulation in PE patients. **Methods:** In this retrospective observational cohort study, we included all adult patients hospitalized with PE at the Cantonal Hospital Baselland (KSBL) between November 2018 and October 2020, where the diagnosis was made within the first twelve hours of their arrival to the emergency department (ED). LASSO regression was employed to identify clinical characteristics associated with delayed anticoagulation initiation. **Results:** A total of 197 patients were included (mean age: 70 years, 54% female). The audit revealed that diagnostic workup was conducted according to guidelines in 57% of cases. Often, D-dimer levels were measured although not strictly necessary (70%). Pretest probability was assessed and documented using the Wells or Geneva score in only 3% of patients, and risk assessment via the Pulmonary Embolism Severity Index (PESI) score was documented in 21% of patients. The median time from ED arrival to CT scan was 120 min (IQR 89.5–210.5), and the median time to anticoagulation initiation was 193 min (IQR 145–277). Factors identified by LASSO associated with delayed anticoagulation included prolonged time from ED arrival to CT scan, the presence of distended jugular veins on examination, ED arrival in the morning, and presenting symptoms of weakness or tiredness. Complementary leg ultrasound was performed in 57% of patients, with 38% of these cases lacking prior clinical examination for deep vein thrombosis. The duration of the anticoagulation treatment was not specified in the discharge report for 17% of patients. A medical follow-up after discharge was recommended in 75% of the patients. **Conclusions:** In conclusion, while the management of PE at the KSBL generally adheres to high standards, there are areas for improvement, particularly in the morning performance, the use of a pretest probability assessment, D-dimer measurement, risk assessment via the PESI score, the performance of complementary leg ultrasounds, clarification of the anticoagulation duration, and follow-up management.

## 1. Introduction

Thromboembolic conditions account for one in four deaths worldwide [1], with pulmonary embolism (PE) being one of the most common acute thromboembolic diseases, alongside myocardial infarction and stroke [2,3]. It is estimated that around 5000 new cases of PE occur annually in Switzerland, indicating that PE is a common and critical condition in Swiss hospitals [4]. Moreover, as the incidence of PE rises with age, PE rates can be expected to continue increasing even further, due to the rapidly aging population in high-income countries, and to significantly impact morbidity, mortality, and healthcare costs in those countries [1,5,6].

The clinical presentation of PE is highly variable, ranging from asymptomatic incidental findings to sudden death [7]. While some symptoms may suggest PE, they are often nonspecific, complicating the diagnostic process [8]. Studies indicate that only about 20% of patients initially suspected of having PE are ultimately diagnosed with the condition [9]. Cardiopulmonary diseases such as acute heart failure, exacerbation of chronic obstructive pulmonary disease (COPD), and pneumothorax or pneumonia are common differential diagnoses that can mask PE [10,11,12]. Given the broad spectrum of symptoms and the potential for misdiagnosis, considering PE as a differential diagnosis is crucial for preventing mortality, as prompt and appropriate treatment significantly reduces the risk of death [13].

Several international, national, and regional guidelines have been developed to standardize the complex management of patients with PE [14,15,16,17,18,19,20,21] (see the overview in the Supplementary Materials). One of the most comprehensive is the European Society of Cardiology (ESC) Guideline for the Diagnosis and Management of Acute Pulmonary Embolism [14]. This guideline, widely adopted across Western Europe, serves as the foundation for many national and regional standards, including those used at the Kantonsspital Baselland (KSBL). At the KSBL, physicians primarily follow diagnostic and treatment algorithms from the licensed “Medstandards” platform of the University Hospital Basel [21], which are based on the ESC guidelines and regularly updated to reflect new evidence. Despite the well-established guidelines, diagnosing PE remains challenging due to its variable symptoms and the need to balance comprehensive evaluation with minimizing unnecessary testing. Furthermore, even after a PE diagnosis is established, managing the condition can be complicated by the various treatment options available.

This audit is part of the QUA-DIT (Quality Evaluation of Hospital Care Through Audits) project, which aims to enhance hospital care and ultimately patient outcomes by evaluating adherence to disease-specific clinical guidelines across key areas of internal medicine at the KSBL [22,23,24,25,26,27,28]. This study aims to retrospectively audit the diagnostic and treatment processes for patients presenting with acute PE at the KSBL between November 2018 and October 2020, assessing the adherence to current guidelines. By identifying weaknesses in the diagnostic workup and treatment processes, the audit seeks to enhance the quality and effectiveness of PE patient management. Additionally, this study will investigate the clinical factors associated with the delayed initiation of anticoagulation, given the evidence that prompt anticoagulation improves outcomes in PE patients [13]. The primary objective of this sub-analysis is to identify preventable factors contributing to delays in treatment.

## 2. Materials and Methods

### 2.1. Design and Setting

This study is a retrospective observational audit conducted on patients with PE admitted to the KSBL, Switzerland, between November 2018 and October 2020.

The KSBL operates across the following three locations within the canton of Baselland: Liestal, Bruderholz Binningen, and Laufen. As a central public teaching hospital with approximately 450 beds, the KSBL serves a population of around 290,000 people [29,30].

### 2.2. Study Population

**Figure 1:**
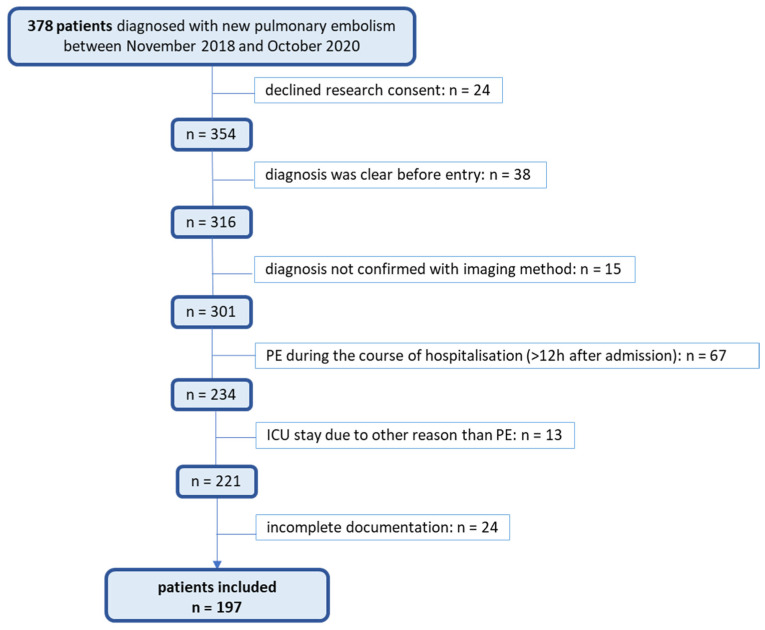
This is a figure. Schemes follow the same formatting. PE = pulmonary embolism; ICU = intensive care unit.

The study population included all patients hospitalized with the International Classification of Diseases (ICD) codes for pulmonary embolism (ICD-10 codes I26.0 for pulmonary embolism with acute cor pulmonale and I26.9 for pulmonary embolism without acute cor pulmonale). Using internal clinical controlling data, 378 hospitalized patients newly diagnosed with PE during this period were identified. All patients included in the analysis were adults. During the study period, 24,556 patients were hospitalized at the medical or surgical ward at the KSBL, with 378 (1.5%) receiving a PE diagnosis during their stay. Each patient’s data were individually reviewed to confirm eligibility based on the study’s inclusion criteria. Adult patients (aged 18 years or older) were included if a new PE was the primary reason for hospitalization and if the diagnosis was confirmed within 12 h of presentation by computed tomography pulmonary angiography (CTPA), scintigraphy, or duplex ultrasound by a specialist. Patients with denied consent or with incomplete/missing diagnostic data were excluded from the study. Ultimately, 197 patients met the study eligibility criteria and were included in the final analysis. The exclusion criteria and the number of patients excluded are detailed in Figure 1.

### 2.3. Data Collection and Analysis

#### Data Collection and Analysis

Data were analyzed using the statistical software R, version 4.0.2. Descriptive statistics for categorical variables were presented as absolute numbers and percentages. Continuous variables were described using means, medians, standard deviations (SDs), and ranges of minimum–maximum values. Statistical distributions were assessed using histograms. To identify independent factors influencing the time between emergency department (ED) admission and the initiation of anticoagulation, the machine learning method LASSO (Least Absolute Shrinkage and Selection Operator) was employed. LASSO was chosen because traditional statistical methods might have been limited by the high number of variables and potential correlations between variables. Since LASSO requires complete data, variables with more than 20% missing values were excluded from the analysis (13 variables). For variables with up to 20% missing data (7 variables), missing values were imputed using the k-Nearest Neighbor (kNN) method with k = 5, available through the “kNN” function in the R package “VIM” [31]. This imputation process resulted in a final dataset comprising 95 variables. The dataset was then randomly divided into a training set (80% of patients, *n* = 144) and a testing set (20% of patients, *n* = 33) to develop and evaluate the LASSO model. The optimal value for the LASSO penalty parameter lambda (λ) was determined using 10-fold cross-validation, as implemented in the R package glmnet. The value λ = 11.86 was selected because it provided the best model fit by minimizing the cross-validation error, leading to a model that effectively balances predictive accuracy and regularization. This process resulted in a subset of important variables retained in the final model. A total of 14 variables with near-zero variance were removed, leaving 81 variables in the final analysis. Ultimately, LASSO regression was performed on the test dataset. A total of 20 variables were identified as potentially influencing the time between ED admission and the start of anticoagulation.

## 3. Results

This study included 197 patients diagnosed with PE within 12 h of presentation at the KSBL during 2018 and 2020. The majority of the patients analyzed in this audit presented to the emergency departments in Liestal (48%) and Bruderholz (40%), with only 12% being treated in Laufen, the smallest of the three hospital locations.

The average patient age was 70 years (range 19–96), with 46% being male. Obesity was prevalent in 38% of patients, with an average BMI of 28.6 kg/m^2^. Additionally, 25% of patients were active smokers, and 90% had relevant pre-existing comorbidities, most commonly hypertension (50%) (see Table 1).

### 3.1. Anamnesis, Clinical Presentation, and Examination

Risk factors for PE were assessed in 58% of cases, while a family history of venous thromboembolism (VTE) was recorded in 43% of cases. The respiratory rate was measured in 86% of patients, and lung auscultation was performed in all cases (100%), revealing abnormalities in 39% of patients. Jugular vein distention was observed in 10% of cases, and signs of deep vein thrombosis (DVT) were checked in 53% of cases, with 30% of cases showing a circumference difference and 20% of cases exhibiting pressure sensitivity in the calf. Electrocardiography (ECG) was performed in 98% of patients, with 17% showing PE-specific changes. PE was initially suspected in 54% of cases. For more information, please see Table 2.

### 3.2. Pretest Probability and Risk Assessment

**Figure 2:**
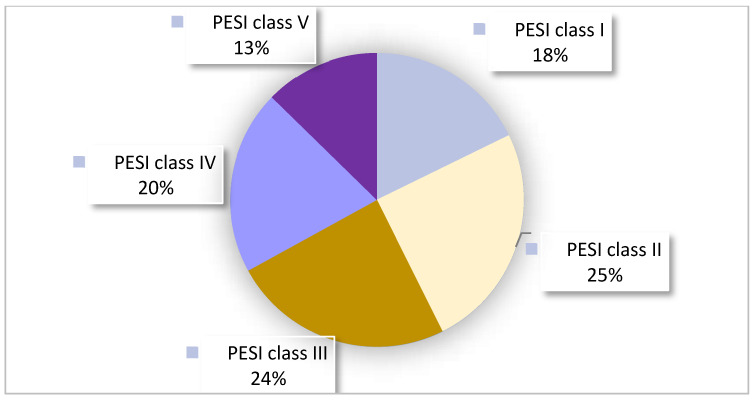
PESI classes used to retrospectively determine the distribution of the Pulmonary Embolism Severity Index (PESI) classes [33]. Class I: less than or equal to 65 PESI points; low 30-day mortality risk from 1% to 6%. Class II: 66–85 PESI points; low mortality risk from 1.7% to 3.5%. Class III: 86–105 PESI points; moderate mortality risk from 3.2% to 7.1%. Class IV: 106–125 PESI points; high mortality risk from 4% to 11.4%. Class V: more than 125 PESI points; high mortality risk from 10% to 24.5%.

Pretest probability using Wells or Geneva scores was calculated and documented in only 3% of patients. Retrospective analysis showed that 51% of patients had a low pretest probability, while 49% of patients had a high probability for PE. The PESI score [32] was documented in 21% of patients, with retrospective recalculations differing in 46% of cases. For more information about pretest probability and risk assessment, please see Table 3. The cohort was fairly distributed across PESI risk classes, with 43% of cases falling into the intermediate–low risk group (Figure 2).

### 3.3. Diagnostic Workup

Diagnostic workup adhered to hospital guidelines in 57% of cases (for more details, see Figure S1, “Overview diagnostic workup suspected PE”, in the Supplementary Materials). D-dimers were measured in 70% of patients, and arterial blood gas analysis was performed in 35% of patients, revealing hypoxemia, hypocapnia, and alkalosis in nearly half of those tested (44%). Cardiac biomarkers (NT-proBNP and troponin) were measured in over 60% of patients, with elevated levels in about half of the patients (53%), indicating right heart strain. Chest radiography was performed in 23% of patients, and CTPA confirmed PE diagnosis in 96% of cases. Right heart strain was detected in 24% of CT scans. Transthoracic echocardiogram (TTE) was performed in 50% of patients, identifying right ventricular distress in 40% of cases. For more information about diagnostic workup, please see Table 4.

### 3.4. Treatments

**Figure 3:**
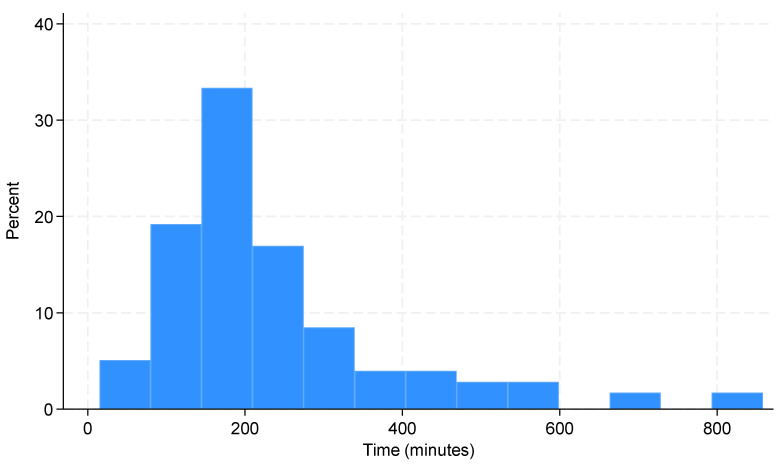
Distribution of time between admission time and anticoagulation administration.

The average time to anticoagulant therapy initiation was 237 min (median 193, IQR 145–277). The distribution of time between admission time and anticoagulation administration is displayed in Figure 3. A loading dose was administered to 16% of patients before CT, and 7% of patients received lysis therapy, primarily via local lysis catheter. Two-thirds of patients received parenteral anticoagulation first, predominantly low-molecular-weight heparin (LMWH), while 39% of patients were started directly on oral anticoagulants, with rivaroxaban being the most commonly prescribed. Anticoagulation duration was generally recommended for 3, 6, or 12 months, or lifelong, though 17% of patients had no specified duration in their discharge reports. During hospitalization, physiotherapy was provided to 58% of patients, while 16% of patients began inhalation therapy. Antibiotics were given to 34% of patients, primarily for urinary tract infections or pneumonia. For more information about treatments, please see Table 5.

### 3.5. Hospitalization and Follow Up

The average length of the hospital stay of the cohort’s PE patients was 6.5 nights. Most patients were treated in the regular ward (70%), while 30% of patients required closer surveillance in the intermediate care unit (IMC) or intensive care unit (ICU), with average ICU stays lasting two nights. After discharge, 88% of patients returned home, 12% of patients were transferred to another care facility, and 21% of patients were rehospitalized within six months. Follow-up care was recommended for 75% of patients, including general practitioner (GP) visits, cardiology, and angiological assessments. Cancer screening was recommended for 28% of patients, with 37% of patients undergoing some form of cancer search during hospitalization. For more information, please see Table 6.

### 3.6. Factors Associated with Delayed Initiation of Anticoagulation (LASSO Analysis)

The LASSO regression analysis identified several factors significantly associated with the delayed initiation of anticoagulation therapy (Table 7), achieving a model R-squared of 0.71, indicating a strong fit. Delays were primarily linked to longer time intervals between presentation and CT scan (+88.85 min), the presence of jugular vein distention during physical examination in the emergency department (ED) (+37.04 min), and the morning entry time (+9.07 min). In contrast, factors associated with the faster initiation of anticoagulation included a performed VTE risk anamnesis (−5.61 min), examination for DVT symptoms (−5.78 min), adherence to diagnostic guidelines (−14.18 min), constant monitoring in the ED (−15.46 min), female sex (−15.56 min), and PE being suspected as the initial diagnosis (−29.44 min). Furthermore, administering a loading dose before the CT scan was associated with the most significant reduction in time to anticoagulation (−31.33 min).

## 4. Discussion

This audit of the management of PE at the KSBL reveals significant opportunities for improvement in diagnostic workup, risk assessment, and treatment protocols. By comparing the current practice to established guidelines, this discussion highlights areas where adherence could be strengthened to enhance patient outcomes and standardize care processes.

The characteristics of the audit cohort align with those reported in larger PE studies. For example, in this audit, the median age at diagnosis was 70 years, 54% of the patients were female, and 50% of patients had hypertension as a comorbidity. In a German study from 2018 with almost one million PE patients, the median age at diagnosis was 72 years, 54% were female, and 43% had hypertension [34]. Furthermore, the overall mortality rate in the audit’s cohort was 5.1%, which is similar to the rate of 5.9%, which an analysis of 24,000 PE patients showed in 2016 [35]. Comparison to the Guidelines: Narrative Discussion

### 4.1. Anamnesis, Clinical Presentation, and Examination

Early diagnosis of PE is crucial for reducing associated morbidity and mortality [13]. The ESC guidelines emphasize the importance of recognizing both predisposing factors and symptoms to accurately determine the clinical probability of PE. Comprehensive history-taking, including assessing personal and family histories of VTE, is vital for making a timely diagnosis [36,37]. However, this audit indicates that only 58% of patients at the KSBL had their risk factors for PE assessed, and just 43% of patients had a documented family history of VTE. The underutilization of DVT examinations, which were performed in only 53% of cases, further exacerbates this issue. Given that PE often originates from DVT, thorough leg examinations should be standard practice to identify early indicators of PE [8]. While nearly every patient received an ECG, as suggested by the guidelines, the lack of documented findings in medical reports limits its utility. It is strongly recommended to describe the ECG findings in the discharge report, even if all findings were normal.

### 4.2. Pretest Probability and Risk Assessment

A further point of discussion is the handling of the pretest probability and Pulmonary Embolism Rule-out Criteria (PERC) score. A pretest probability was only documented in five of the cohort patients (3%). According to the ESC guidelines, the pretest probability does not have to be assessed with the Geneva or Wells score; clinical judgment is sufficient for experienced physicians. However, to make decisions more understandable for the medical staff taking over after the emergency care, it would be beneficial to document the scores or the clinical judgments in the medical records. Furthermore, this assessment is crucial for determining the path of the further diagnostic workup (i.e., conduct D-dimers testing first or proceed directly to CTPA). Hence, if not evaluated, there is a high probability that the diagnosis will not be performed according to the current guidelines.

The PE Rule-out Criteria (PERC) score was retrospectively calculated for all eligible patients, revealing that 3% of patients would not have qualified for further testing based on their negative PERC scores. Although the PERC score’s utility remains debated, with some studies indicating higher false-negative rates, it is still a valuable tool when applied appropriately [38,39,40]. The findings of this audit align with international concerns about the underuse of these tools, suggesting that implementing a flowchart in the emergency department (ED) could improve documentation and reduce unnecessary testing [41].

Risk assessment using the PESI score was similarly underutilized, with only 21% of patients having documented scores. Additionally, retrospective recalculations showed discrepancies in 46% of these cases, indicating inconsistencies in risk stratification. Documenting the specific criteria used in PESI score calculations could improve clarity and ensure appropriate treatment decisions. Moreover, in our recent publication, we found that the PESI score was a strong predictor for length of hospital stay (LOHS), mortality, and rehospitalization [42]. The PESI score is an essential parameter for determining patient management strategies (e.g., outpatient treatment and surveillance). Therefore, whenever it is not calculated, the patient is at risk of being under- or overtreated.

### 4.3. Diagnostic Workup

The diagnostic workup for PE in this study was largely in line with hospital guidelines; however, there were areas for improvement. Adherence to guidelines in terms of diagnostic workup was observed in 57% of cases. D-dimer testing, a crucial tool in the initial assessment of suspected PE, was conducted in 70% of patients, demonstrating high sensitivity (99%). Only 1 patient out of the 138 tested returned a false-negative result, consistent with the Cochrane Database of Systematic Reviews from 2016, which confirmed the reliability of D-dimer as a diagnostic tool [43]. Although the audit showed that the performance of the D-dimer testing is already high, the current guidelines suggest that the use of age-adjusted D-dimer cut-off values in people aged >50 years may further improve the performance [14,44]. This audit showed that the standard D-dimer cut-off value was preferred rather than an age-adjusted one. The failure to adopt this adjustment in clinical practice may lead to the overuse of imaging modalities, exposing patients to unnecessary risks and increasing healthcare costs. Implementing age-adjusted cut-offs should be prioritized to enhance the diagnostic accuracy and efficiency of the PE workup.

CRP testing was performed in almost all of the patients of the cohort (99.5%), likely due to the need to differentiate between pulmonary embolism (PE) and other conditions like pneumonia, which can present with similar symptoms. CRP was elevated in nearly 90% of the patients and, in most cases, it was attributed to PE, with only a small number of patients being evaluated for coexisting infections and receiving antibiotics. The literature lacks consensus on whether CRP elevation is a consequence of PE or a contributing factor [45,46]. The high incidence of CRP elevation in PE patients was observed over a decade ago, with some studies exploring CRP’s potential role in excluding PE, either alone or alongside pretest probability scores [47,48]. However, these studies produced controversial results, and the topic lost attention until 2018, when research suggested that elevated CRP from acute inflammation could trigger VTE, even after accounting for acute infections. This led to the hypothesis that non-infectious conditions, such as cancer, obesity, trauma, or surgery, might elevate CRP levels and thus act as PE risk factors [49]. Further research is needed to clarify the relationship between CRP and PE.

All recommended PE diagnostic imaging methods (i.e., CT, scintigraphy, and CUS combined with PE-specific symptoms) were used at the KSBL, with CTPA being the most common. The average time from ED admission to CT scan was just two and a half hours (162 min; median time 2.0 h). In a 2011 study, Smith et al. found that the median time between ED arrival to CT diagnosis was 2.4 h [50], while a 2018 study by Willoughby et al. showed a median time of 3.1 h [51]. This suggests that the diagnostic speed at the KSBL is comparable to, or slightly faster than, other hospitals.

The use of ABG testing was inconsistent, with no clear criteria guiding its application. This audit found that 62% of patients with respiratory symptoms did not receive an ABG test, even when their oxygen saturation was low. Although ABG testing is not mandatory according to the guidelines due to its non-specificity, establishing clear rules for its use could improve diagnostic efficiency and resource management [14,52]. Implementing such rules might not only improve the overall performance but also save resources, as this issue likely affects the diagnostic process for other conditions as well. Supporting this view, previously published Swiss audits of the management of acute respiratory conditions showed that there is room for improvement in Swiss hospitals regarding ABG diagnostics [25,27,53].

In addition, the current guidelines accept the use of compression ultrasonography (CUS) of the legs to confirm PE in patients with DVT and PE symptoms. However, the role of CUS after a PE diagnosis is made remains unclear and is not discussed in the current ESC guidelines [14]. At the KSBL, the decision to perform CUS after a PE diagnosis seems to be physician-driven, and no clear standards exist. A brief internal inquiry revealed differing opinions among in-house angiologists regarding the necessity of CUS in this context, reflecting a lack of consensus. The audit showed this ambiguity, as not all patients with DVT signs received an ultrasound, while some without any calf complaints underwent the procedure. Considering the high prevalence of concomitant DVT in PE patients, it is economically efficient to reserve CUS for those who have a PE diagnosis and present with painful and swollen legs [54]. If a DVT is diagnosed in these patients, a therapeutic trial with compression stockings can be started to potentially achieve symptom reduction [55]. For patients without these specific symptoms, additional DVT diagnosis via CUS does not typically alter the treatment plan, suggesting that the resources might be better allocated to other necessary procedures. The authors advocate for this issue to be addressed in future revisions of the ESC guidelines.

Once the PE diagnosis is made, it is essential to decide whether the PE was provoked or not, as this is a determinant for the further treatment plan [14,16]. At this point, it should be noted that the newest guidelines no longer support the terms “provoked” and “unprovoked” PE. Instead, they refer to the “presence of transient or reversible risk factors” and the “absence of identifiable risk factor” to determine the recurrence risk [14]. Despite this change, the terminology of “provoked” and “unprovoked” remains very common in the vocabulary of the physicians and continues to be used interchangeably with the new terms.

In the audit cohort, PE was categorized as provoked if this label appeared anywhere in the patient’s medical reports. Using this method, 61% of the PEs were classified as unprovoked and only 39% of PEs were classified as provoked. Compared to other studies, the number of provoked PEs appears to be low. In an analysis of the International Cooperative Pulmonary Embolism Registry (ICOPER), no apparent risk factor or coexisting disorders could be found in up to 20% of PE patients [56]. In the PIEDOD II study from 2007, at least one risk factor was discovered when specifically searched for in over 90% of the patients [57]. However, it is difficult to directly compare these numbers, as there is no universal definition for the term “risk factors”. A different cluster of factors was analyzed in every study, sometimes including common factors such as gender or obesity, and sometimes excluding them. Given the importance of risk factors in determining the duration of anticoagulation therapy, the KSBL could place more emphasis on identifying these factors. Accurate risk factor identification is critical because it influences the treatment duration: lifelong therapy is recommended for patients with idiopathic PE or lasting risk factors, while a 3–6-month course is typically sufficient for provoked PEs associated with transient factors [16]. This is particularly important because anticoagulation therapy carries a risk of severe side effects, making it essential to avoid the unnecessary prolongation of treatment [58,59,60].

### 4.4. Treatments

The ESC guidelines recommend initiating anticoagulant therapy in cases where PE is highly probable and bleeding risk is low, even before diagnostic confirmation [14]. However, in this audit, pre-emptive anticoagulation was administered to only 16% of the patients. The administration was mainly conducted in the ED by the emergency physicians even though nearly half of the patients were referred to the hospital by another doctor (mostly their GP), often with an already high PE suspicion. As the GPs usually know their patients well and can quickly assess the risk of bleeding, they could be more courageous in administrating a loading dose before referring the patient to the hospital. Willoughby et al. made similar observations in their 2018 study [51]. As research on this topic is still scarce, further analyzing whether the administration of a loading dose indeed significantly changes the patient’s outcome or not might be interesting.

While a few studies analyzed the time between admission to the ED and CT scan in PE patients, no similar analysis of the time between ED entry and the anticoagulation initiation in a comparable patient group could be found. In the audit, the mean time between admission and the start of anticoagulation was 4 hours (237 min).

Rivaroxaban was the most commonly used oral anticoagulant, likely due to its ease of use, including immediate administration without the need for bridging therapy [61]. Interestingly, dabigatran was not prescribed, potentially due to its more complex dosing requirements. Lysis therapy was administered to 7% of patients, with a preference for local lysis using the EKOS^®^ catheter. However, the KSBL’s limited ICU capacity sometimes necessitated patient transfers, which may have led to an underestimation of the number of patients receiving lysis therapy in this audit.

In about 17% of cases, the recommended minimum duration of the anticoagulant treatment was not described in the discharge report. This means that the GPs who did not witness the acute phase and depended on the discharge report for information had to decide on the duration and further course of action on their own. This situation can be more challenging for GPs compared to hospital doctors who directly manage the acute phase and tailor the treatment. Without clear guidance, there is a risk that patients might receive anticoagulation for too short or too long a period. Hence, it is strongly recommended that the minimum time of the anticoagulant therapy is specified in the discharge report.

### 4.5. Hospitalization and Follow-Up

The average length of stay of the cohort’s PE patients was 6.5 nights. In the annual report of the Swiss Federal Office Of Public Health (FOPH), the median length of stay for patients hospitalized in 2019 with PE was 6 and 5 days (for PE with acute cor pulmonale and for PE without acute cor pulmonale, respectively) [4,30]. This indicates that PE patients in our cohort were hospitalized for a slightly longer duration compared to the general PE population in Switzerland.

Bed rest in VTE patients is considered old-school and no longer recommended, as it showed no benefit in studies [62]. That applies even if a floating thrombus is seen in the leg ultrasound; no clear recommendations exist for saddle thrombi in the lung [19]. In the audit cohort, almost one-fifth (18%) of the patients were prescribed bed rest after diagnosis, and this percentage appears comparatively high. It could be partly explained by the use of lysis therapy, which has a high risk of severe bleeding when falling, but, for many patients, no clear reason was apparent. Neither the ESC guideline nor the Medstandards provide guidance on the prescription of bed rest in PE patients [14,21]. It might be beneficial if the next version of the guideline addresses this issue so to avoid patients being unnecessarily restricted to bed rest.

A critical point in the follow-up care for PE is cancer screening, which remains a controversial topic among experts [63]. Since undiagnosed malignancy can potentially lead to an unprovoked PE, screening for cancer might be considered for patients with unexplained PE. In the cohort, cancer screening was conducted, recommended, or planned for approximately half of the patients (48%). Ultrasound of the abdomen and lab analysis of cancer markers in the blood were the most frequently used diagnostic tools. However, there was a lack of standardized criteria for screening, and decisions appeared to be predominantly physician-driven. In some cases, cancer screening was planned for patients with PE classified as provoked, which, in the absence of additional cancer indicators, led to unnecessary tests and avoidable costs. The current ESC guidelines do not address cancer screening in detail, leaving room for future updates to clarify recommendations. Simple, quick assessments, such as asking about previous colonoscopies or recent gynecological/urological check-ups, could be easily integrated into routine care at no additional cost. Additionally, basic physical examinations, such as breast or prostate exams, could be performed by ward physicians with minimal effort. For more specialized tests, adherence to national age- and gender-specific cancer screening guidelines is recommended [20]. Similarly, thrombophilia screening in PE patients is briefly covered in the guidelines and should only be carried out when there is a clear indication [20].

### 4.6. Factors Associated with Delayed Initiation of Anticoagulation (LASSO Analysis)

The LASSO analysis confirmed that PE as an initial suspicion diagnosis (loading dose even before CT scan) and the presence of “typical” VTE symptoms (e.g., new leg swelling and tachypnea or dyspnea) were associated with a faster start of the anticoagulation treatment. On the other hand, variables implying that PE was not the main suspected diagnosis (e.g., weakness/tiredness in anamnesis, jugular vein distention in examination, and a long time elapsed until the start of CT scan) led to a slower anticoagulation initiation. Although JVD is recognized as a sign of PE, it is not specific to it and can be associated with other cardiac conditions, such as heart failure. The reason why JVD is associated with a delay in the anticoagulation initiation may result from clinicians considering alternative diagnoses, which could have postponed the anticoagulation treatment until the confirmation of PE through imaging (CT scans). This emphasizes the critical need to consider PE as a potential diagnosis in patients presenting with relevant symptoms.

The finding that anticoagulation was initiated more quickly in patients with a positive ICU status (meaning in patients who would not refuse an ICU stay) may reflect a bias toward treating younger, healthier patients more aggressively, as they present fewer differential diagnoses. An unexpected finding was the slight delay associated with morning entry times in the ED, which may be due to reduced staffing levels or more comprehensive patient evaluations during quieter morning hours. While the delay was minimal, understanding the reasons behind it could help optimize workflow in the ED.

Research suggests that the continued stigmatization of people with mental illness affects their medical care. For example, O’Rourke et al. showed in 2008 that psychiatric illness was associated with delayed diagnosis of esophageal cancer and, thereby, advanced disease at the time of diagnosis [64]. This problem is also conceivable in other patients who might be stigmatized by society for various reasons, such as severely obese people or infirm elderly people, which is why the audit data were also checked for these groups. Interestingly, the data indicated that infirm elderly individuals, people with obesity, and those with mental illnesses were not treated differently from other patients at the KSBL, suggesting that stigmatization did not impact their care. Additionally, the analysis found that women were anticoagulated faster than men, which is a surprising result given that previous research has shown gender disparities in the treatment of other conditions, typically favoring men (e.g., myocardial infarction [65] or COPD [66]). However, similar observations have been made in other studies. For example, a 2015 study by Bach et al. found that the time to diagnosis was prolonged in male PE patients [67]. A possible explanation for this pattern could be that, in Western society, a young smoking woman under birth control is often portrayed as a typical PE patient, leading to a quicker diagnosis in women, but further research is needed to validate this hypothesis. Finally, adherence to diagnostic guidelines was associated with faster anticoagulation initiation, reinforcing the importance of following established protocols. Given that prompt and appropriate therapy is known to reduce mortality in PE patients [13], following these guidelines is likely to improve patient outcomes.

### 4.7. Strength, Limitations, and Further Research

Among the limited audits previously conducted on the management of PE patients, the majority focused on specific aspects of the process, for instance, the use of clinical decision rules [68] or treatment strategies [69], or the examination of single subsegmental PEs [70]. This is the first audit of its extent, analyzing the whole diagnostic workup and treatment process. It can form the groundwork for further similar audits and enable cross-hospital comparisons, which could provide valuable insights into the strengths and areas for improvement at the KSBL. The characteristics of the audit cohort align with those reported in larger PE studies, enhancing the generalizability of our findings.

The retrospective nature of the audit introduces potential biases. We assumed that any information not documented in the medical records was not measured, performed, or analyzed by the treating medical staff. Due to incomplete patient records, this approach might not fully reflect reality, and the audit might slightly underestimate the actual figures, making some aspects of the performance at the KSBL look poorer than reality (e.g., determination of the pretest probability).

The exclusion criterion of “delayed diagnosis of PE (>12 h after admission)” was used to avoid including PEs that developed after admission. Including these PEs would have falsified the audit’s results, as there were most likely no PE signs at presentation time, meaning that the diagnostic would not have been performed according to the guidelines. The 12-h cut-off was chosen based on a definition by Smith et al. in 2011, who used this threshold to differentiate between early and delayed PE diagnoses [50]. However, the exclusion of delayed diagnosis of PE may have resulted in the exclusion of some cases where PE was present at presentation but particularly difficult to diagnose.

It is also important to note that the study does not account for PEs that went undetected entirely, including those identified only post-mortem or resolved without diagnosis. This limitation means that the audit cannot address the reasons for missed diagnoses or account for cases where PE was never identified. A limitation of our analysis is the potential bias introduced by the LASSO regression, which shrinks coefficients and may underestimate the true effect sizes of predictors, particularly when variables are correlated. However, this bias improves generalization by reducing model variance, and LASSO analysis has the advantage of providing improved prediction accuracy and variable selection. This study primarily focused on the indications for anticoagulation in acute pulmonary embolism, and while we recognize the importance of bleeding risk assessments, our analysis did not incorporate these factors, as current guidelines allow for clinical judgment based on individual risk assessments at the time of initiation. Future research should investigate the integration of bleeding risk assessments into the clinical workflow, particularly after the acute management phase.

Further research should focus on exploring gender disparities in clinical management, as understanding how presentation, treatment decisions, and outcomes may differ between men and women can provide critical insights for improving personalized care and ensuring equitable treatment across all patient groups.

## 5. Conclusions

Overall, the audit showed that the management of PE patients at the KSBL between November 2018 and October 2020 was of a high standard. Confirmation of the suspicion diagnosis by imaging and anticoagulation treatment were typically prompt. However, not all patients were treated according to the current internal guidelines, leaving room for improvement.

Key recommendations for improvement based on the audit’s findings include the following:

- Identifying and documenting risk factors, family history, and signs of DVT in medical records when PE is suspected.
- Evaluating the pretest probability for suspected PE patients, measuring D-dimers only when the probability is low, and using age-adjusted cut-offs to improve the testing efficiency. For high-pretest-probability patients, direct CTPA should be considered.
- Documenting ECG findings comprehensively in medical records to maximize the benefit of ECG recordings.
- Establishing clear criteria for ABG testing in the ED, limiting its use to patients with respiratory symptoms to save resources.
- Using PESI or sPESI scores for risk assessments; measuring troponin and pro-BNP levels in intermediate-risk patients for further classification.
- Avoiding bed rest for patients with acute PE.
- Performing leg ultrasonography only in patients with leg symptoms, as additional asymptomatic DVT diagnoses have limited impact on treatment decisions.
- Identifying and documenting identifiable risk factors for PE, which is crucial for treatment planning.
- Defining the minimal duration of anticoagulation treatment in discharge reports to prevent under- or overtreatment.
- Planning follow-up examinations, such as cancer screening and thrombophilia testing, with careful consideration of their benefits.
- Encouraging physicians to use anticoagulation loading doses more readily in patients with a high suspicion of PE and a modest bleeding risk.

## Figures and Tables

**Table 1:** Baseline characteristics.

Characteristics	All (*n* = 197)	Missing, *n* (%)
Age at diagnosis (years), mean (range)	70 (19–96)	--
Gender (male)	90 (46%)	--
Body mass index (BMI), mean (SD) (kg/m^2^)	28.6 (±6.40)	21 (10.6%)
Obesity (BMI ≥ 30)	72 (38%)	7 (3.5%)
Smoking Status	111 (56.4%)	86 (43.6%)
Lifelong non-smoker	48 (43%)	--
Current smoker	28 (25%)	--
Former smoker	35 (32%)	--
Comorbidities	178 (90%)	--
Active cancer	12 (6%)	--
Cardiovascular disease	69 (35%)	--
Chronic lung disease	35 (18%)	--
Hypertension	98 (50%)	--
Mental health disorder	49 (25%)	--
Severe renal insufficiency	21 (11%)	--

SD = standard deviation; BMI = body mass index.

**Table 2:** Anamnesis, clinical presentation, and examination.

Medical History-Taking	All (*n* = 197)	Missing/Not Performed, *n* (%)
Risk factor anamnesis asked	115 (58%)	--
Family history for VTE asked	85 (43%)	--
Smoking status cleared	111 (56%)	--
B-symptoms asked	41 (21%)	--
Clinical examination		
Respiratory rate measured	169 (85.7%)	28 (14.2%)
DVT symptoms checked	105 (53%)	92 (46.7%)
Pressure pain in the calf	20 (20%)	98 (49.7%)
Circumference difference	31 (30%)	92 (46.7%)
Jugular vein assessed	159 (81%)	38 (19.3)
Jugular vein distention	16 (10%)	38 (19.3)
Lung auscultation	197 (100%)	--
Rales	77 (39%)	--
Initial suspicion diagnosis PE	107 (54%)	--
Electrocardiography assessments		
ECG performed	193 (98%)	4 (2%)
ECG analysed in discharge report	146 (76%)	4 (2%)
PE specific abnormalities described	25 (17%)	

ICU = intensive care unit; VTE = venous thromboembolism; DVT = deep vein thrombosis.

**Table 3:** Pretest probability and risk assessment.

Pretest Probability	All (*n* = 197)
Pretest probability calculated (Geneva or Wells score)	5 (3%)
Geneva score calculated retrospectively:	
Low pretest probability (0–2 P)	101 (51%)
High pretest probability (≥3 P)	96 (49%)
Risk assessment with PESI	
PESI score calculated and documented	41 (21%)
Retrospectively differently calculated than in the report	19 (46%)

**Table 4:** Diagnostic workup.

PE-Specific Laboratory	All (*n* = 197)
Diagnostic according to guidelines	113 (57%)
D-dimers measured	138 (70%)
D-dimers unnecessarily measured	59 (70%)
D-dimers wrongly not measured	21 (25%)
Troponin T hs measured	129 (65%)
NT-proBNP measured	150 (76%)
Arterial blood gas analysis performed in the ED	68 (35%)
No ABG despite respiratory problems	97 (62%)
Performed diagnostic imaging	
Chest radiography	46 (23%)
Computed tomography pulmonary angiography	189 (96%)
Right ventricular stress signs	46 (24%)
Time between admission and CT, mean (SD) in minutes	162 (±110)
Scintigraphy	6 (3%)
Diagnosis only by compression ultrasound	4 (2%)
Transthoracic echocardiogram	98 (50%)
Ultrasound leg	
Ultrasound leg performed	113 (57%)
Thrombosis found	83 (73%)
CUS performed	113 (57%)
CUS because of symptoms	51 (45%)
CUS despite legs not examined	43 (38%)
No CUS despite symptoms or signs	10 (12%)

PE = pulmonary embolism; NT-proBNP = N-terminal pro-b-type natriuretic peptide; ABG = arterial blood gas; SD = standard deviation; ED = emergency department; CT = computed tomography; CUS = compression ultrasound.

**Table 5:** Treatments.

Anticoagulant Treatment	All (*n* = 197)
Time between entry and anticoagulation, mean (SD) in minutes	237 (±150)
Loading before CT	31 (16%)
Lysis therapy	13 (7%)
Systemic lysis	3 (23%)
Local lysis (EKOS^®^)	10 (77%)
Parenteral anticoagulation	120 (61%)
Oral anticoagulation right from the beginning	77 (39%)
Oral anticoagulation before discharge	186 (94%)
Maximum duration anticoagulation:	(*n* = 187) *
3 months	42 (21%)
6 months	45 (23%)
12 months	7 (4%)
Lifelong	56 (28%)
As long as a risk factor is present	3 (1.5%)
No timeframe in the discharge report	34 (17%)
Non-anticogulant treatment	(*n* = 197)
Physiotherapy	114 (58%)
New inhalation therapy	32 (16%)
Antibiotic therapy during hospitalisation	67 (34%)

SD = standard deviation; CT = computerized tomography; EKOS = EkoSonic^®^ System. * 10 patients died during hospital stay.

**Table 6:** Hospitalization and follow up.

Hospitalization	All (*n* = 197)
Length of stay, median (range)	6.5 (1–32)
Non-stop regular ward	138 (70%)
Surveillance at IMC or ICU	59 (30%)
IMC stay	23 (12%) *
Length of IMC stay, mean (SD) in nights	1.4 (±0.6)
ICU stay	37 (19%) *
Length of ICU stay, mean (SD) in nights	2.0 (±1.1)
Initial bed rest	36 (18%)
In-hospital death	10 (5.1%)
Cancer search	
Cancer search performed at all (recommended and planned)	94 (48%)
Cancer search despite provoked PE	19 (10%)
No consciously performed cancer search	30 (29%)
Incidental finding of initial PE-CT control	16 (8%)
Follow up	(*n* = 187)
Suggested follow-up inspection	140 (74.9%)
Coagulation assessment recommended	30 (16%)
Cancer search recommended	56 (29.9%)

IMC = intermediate care unit; ICU = intensive care unit; SD = standard deviation; PE = pulmonary embolism; CT = computed tomography. * one patient was in the IMC first and then the ICU.

**Table 7:** LASSO regression—coefficients of predictors on time between admission and the start of anticoagulation.

Variable	Estimate (in Min)
Time between presentation and CT scan	+88.85
Jugular vein distention present in examination in the ED	+37.04
Weakness or tiredness as a symptom	+23.24
Entry time, morning	+9.07
Rheumatic disease in medical history	+0.85
Entry time, evening	+0.30
BMI	−0.54
Tachypnea (>20/min) as a symptom	−1.36
Dyspnea as a symptom	−1.47
ABG performed in the ED	−1.63
Hypertension in the ED	−2.69
Risk factors anamnesis performed	−5.61
DVT symptoms checked in examination in the ED	−5.78
New leg swelling as a symptom	−10.67
Diagnostic approach was according to internal guidelines	−14.18
Constant monitoring in the ED (except the imaging process)	−15.46
Female sex	−15.56
ICU status positive (patient did not decline an ICU stay)	−21.85
PE was initially suspected as a diagnosis	−29.44
Patient received loading dose before CT scan	−31.33

CT = computerized tomography; ED = emergency department; BMI = body mass index; ABG = arterial blood gas; ICU = intensive care unit.

## Data Availability

The data presented in this study can be made available from the corresponding author upon reasonable request. The data are not publicly available due to restrictions pertaining to data privacy.

## Supplementary Materials

The following supporting information can be downloaded at: https://www.mdpi.com/article/10.3390/jcm13206103/s1, Figure S1: Overview diagnostic workup suspected PE; Table S1: List of pulmonary embolism guidelines.
