# Supplementary material for: Pulmonary Embolism Management Audit and Machine Learning Analysis of Delayed Anticoagulation in a Swiss Teaching Hospital
Source: J Clin Med. 2024 Oct 13;13(20):6103. doi: 10.3390/jcm13206103 (PMC11508303; doi:10.3390/jcm13206103)
Supplement: Supplementary file 1 [file jcm-13-06103-s001.zip › jcm-3249245-supplementary.pdf]

SUPPLEMENTARY MATERIAL

Table S1. List of pulmonary embolism guidelines

Guideline	Year	Country
2019 ESC Guidelines for the diagnosis and management of acute pulmonary embolism of the European Society of Cardiology (ESC) [14]	2019	Europe
American Heart Association (AHA): Management of Massive and Submassive Pulmonary Embolism, Iliofemoral Deep Vein Thrombosis, and Chronic Thromboembolic Pulmonary Hypertension [15]	2011	United States of America
Antithrombotic Therapy for VTE Disease CHEST Guideline and Expert Panel Report [16]	2016	
American Society of Haematology 2020 guidelines for management of venous thromboembolism: treatment of deep vein thrombosis and pulmonary embolism [17]	2020	
NICE Guideline on venous thromboembolic diseases: diagnosis, management and thrombophilia testing [NICE Guideline No. 15897] [18]	2020	United Kingdom
S2k-Leitlinie zur Diagnostik und Therapie der Venenthrombose und der Lungenembolie [19]	2015	Germany
MediX-Guideline Thormboembolie [20]	2020	Switzerland
medStandards University Hospital Basel [21]	2021	Switzerland

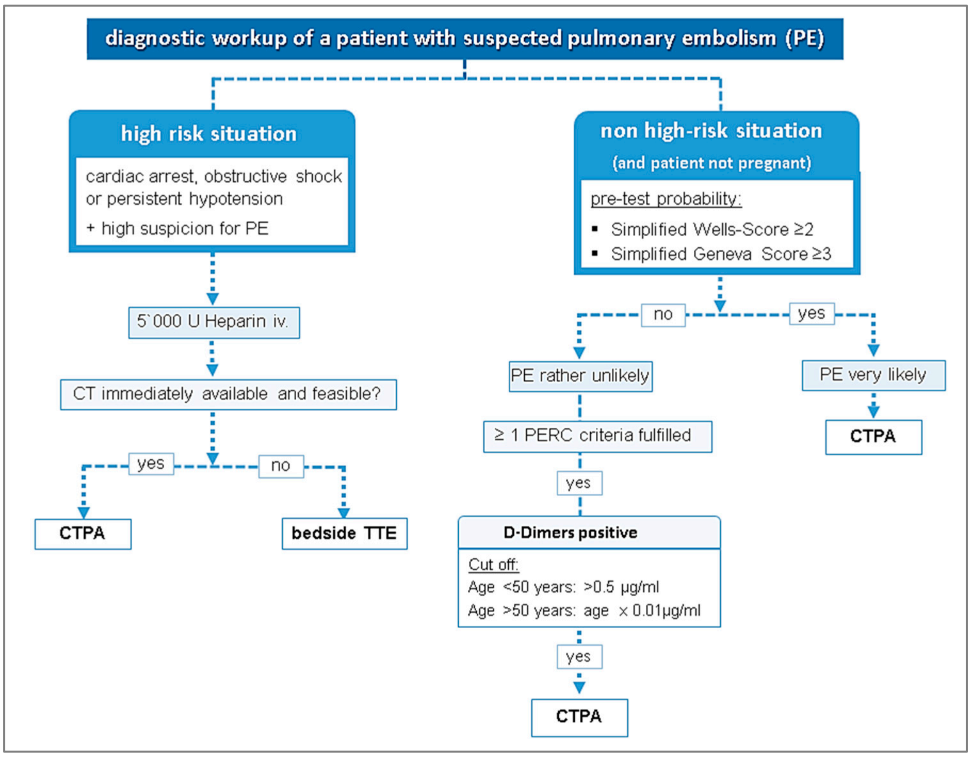

Figure S1. Overview diagnostic workup suspected PE
